# Upper gastrointestinal bleeding due to peptic ulcer disease is not associated with air pollution: a case-crossover study

**DOI:** 10.1186/s12876-015-0363-6

**Published:** 2015-10-14

**Authors:** Samuel Quan, Hong Yang, Divine Tanyingoh, Paul J. Villeneuve, David M. Stieb, Markey Johnson, Robert Hilsden, Karen Madsen, Sander Veldhuyzen van Zanten, Kerri Novak, Eddy Lang, Subrata Ghosh, Gilaad G. Kaplan

**Affiliations:** 1 Departments of Medicine and Community Health Sciences, University of Calgary, 3280 Hospital Drive NW, Room 6D56, Calgary, AB T2N-4N1 Canada; 2 Departments of Emergency Medicine, University of Calgary, Calgary, T2N-4N1 Alberta Canada; 3 Population Studies Division, Health Canada, Vancouver, British Columbia V6C 1A1 Canada; 4 Air Health Science Division, Health Canada, Ottawa, K1A 0K9 Ontario Canada; 5 Department of Health Sciences, Carleton University, Ottawa, Ottawa, K1S 5B6 Ontario Canada; 6 Department of Medicine, University of Alberta, Edmonton, T6G-2R7 Alberta Canada

**Keywords:** Air pollution, Peptic ulcer disease, Upper gastrointestinal bleeding, Particulate matter, Nitrogen dioxide

## Abstract

**Background:**

Recent studies have demonstrated an association between short-term elevations in air pollution and an increased risk of exacerbating gastrointestinal disease. The objective of the study was to evaluate if day-to-day increases in air pollution concentrations were positively associated with upper gastrointestinal bleeding (UGIB) secondary to peptic ulcer disease (PUD).

**Methods:**

A time-stratified case-crossover study design was used. Adults presenting to hospitals with their first UGIB secondary to PUD from 2004–2010 were identified using administrative databases from Calgary (*n =* 1374; discovery cohort) and Edmonton (*n =* 1159; replication cohort). Daily concentrations of ozone, nitrogen dioxide, sulfur dioxide, carbon monoxide, and particulate matter (PM_10_ and PM_2.5_) were estimated in these two cities. Conditional logistic regression models were employed, adjusting for temperature and humidity. Odds ratios (OR) with 95 % confidence intervals (CI) were expressed relative to an interquartile range increase in the concentration of each pollutant.

**Results:**

No statistically significant associations were observed for any of the individual pollutants based on same-day, or 1-day lag effects within the Calgary discovery cohort. When the air pollution exposures were assessed as 3-, 5-, and 7-day averages, some pollutants were inversely associated with UGIB in the discovery cohort; for example, 5-day averages of nitrogen dioxide (OR = 0.68; 95 % CI: 0.53–0.88), and particulate matter <2.5 μm (OR = 0.75; 95 % CI: 0.61–0.90). However, these findings could not be reproduced in the replication cohort.

**Conclusion:**

Our findings suggest that short-term elevations in the level of ambient air pollutants does not increase the incidence of UGIB secondary to PUD.

**Electronic supplementary material:**

The online version of this article (doi:10.1186/s12876-015-0363-6) contains supplementary material, which is available to authorized users.

## Background

Upper-gastrointestinal bleeding (UGIB) secondary to peptic ulcer disease (PUD) is a serious medical condition that is associated with substantial morbidity, high healthcare costs, and decreased quality of life [1, 2]. Patients suspected to have UGIB secondary to PUD require immediate medical attention [3]. Despite advances in management, the risk for mortality among patients with this condition ranges from 2.2 % upward to 14 % [4–9]. The primary risk factors for PUD are non-steroidal anti-inflammatory drugs (NSAIDs) and *Helicobacter pylori*. However, a significant proportion of PUD is not explained by these risk factors [10, 11]. It is important to identify other risk factors in order to mitigate the burden associated with PUD that is not caused by NSAIDs and *Helicobacter pylori*.

Epidemiologic and historical data suggest that PUD is a relatively modern disease of industrialized nations [12]. Autopsy data showed virtually no cases of death secondary to PUD in the early 1800’s, but by the mid 1800’s PUD became recognized as a major contributor to mortality in Westernized nations like North America and England [12]. Aspirin was only introduced in the early 1900’s and thus, the epidemic rise of PUD is hypothesized to have been caused by a virulent strain of *Helicobacter pylori* [12]. During the 20^th^ century the incidence of PUD has decreased. This is due, in part, to the identification of NSAIDs and *Helicobacter pylori* as the key risk factors for PUD. However, other contributing factors may explain the epidemiological patterns that describe the rise and fall of PUD over the past 200 years.

Ambient air pollution contributes to the development of a number of medical conditions [13]. Air pollution has been associated with an increased risk of cardiovascular disease (e.g. myocardial infraction) [14, 15], respiratory disease (e.g. chronic obstructive pulmonary disease, asthma) [16–19], stroke [20], cancer [21], and premature mortality [22]. Recent discoveries suggest that air pollutants affect the gastrointestinal tract and contribute to the development of inflammatory bowel disease [23, 24], appendicitis [25, 26], and non-specific abdominal pain [27]. Furthermore, exposure to air pollutants has been shown to affect the physiology of the gastrointestinal tract by altering intestinal permeability, microbial composition and diversity, and intestinal immunity, which may help promote the development of gastric ulcers [28–31]. Finally, one study has demonstrated a correlation between death from peptic ulcer disease and exposure to air pollution [32].

Thus, we hypothesize that exposure to acute air pollution may be a contributing factor in the initiation of an UGIB secondary to PUD.

## Methods

### Study population

Incident cases of UGIB secondary to PUD were identified using hospital administrative databases. The province of Alberta provides medical care services to all eligible residents, such that 99 % of the population is insured [33]. The discharge abstract databases (DAD) contain information for all hospital discharges within the province of Alberta. Furthermore, DAD contains high quality information including the date of hospital admission and discharge; the patient’s age, sex, and place of residence; and up to 25 diagnostic codes classified using the International Classifications of Diseases version 10 (ICD-10) system. Charlson comorbidities [34] (excluding PUD) were extracted.

We identified all adult (≥18 years) residents of Calgary and Edmonton hospitalized with a newly diagnosed UGIB secondary to PUD (ICD-10: K2X.0, K2X.2, K2X.4, K2X.6, where X = 5–8) from 2004 to 2010. The ICD-10 codes for UGIB secondary to PUD were previously validated with a positive predictive value of 85.2 % and sensitivity of 77.1 % [1]. We limited the analyses to patients whose hospital stay was attributed to UGIB secondary to PUD (i.e. UGIB secondary to PUD was coded in the primary position in the discharge abstract). To remove subsequent episodes of UGIB secondary to PUD, unique personal health numbers were used to identify multiple admissions for the same patient. A two year washout period (i.e. no prior codes for UGIB secondary to PUD) was used to confirm that our study population only contains incident cases of UGIB secondary to PUD hospitalized within the years of 2004 to 2010 within the city of Calgary (*n =* 1374) and Edmonton (*n =* 1159).

We first tested our hypothesis using the population residing within the city of Calgary (i.e. discovery cohort), Alberta. Subsequently, the analysis was replicated using the population living in Edmonton, Alberta (i.e. replication cohort). Administrative data in both of these cities was collected by the same agency, Alberta Health.

### Air pollution and meteorological data

Environment Canada’s National Air Pollution Surveillance Network monitors air pollutants in locations across Canada. These monitors record hourly exposure to selected air pollutants. We employed data on sulfur dioxide (SO_2_), nitrogen dioxide (NO_2_), carbon monoxide (CO), and ozone (O_3_), as well as particulate matter with aerodynamic diameter of ≤10 μm (PM_10_) and ≤2.5 μm (PM_2.5_). Hourly measurements of the individual pollutants were averaged across multiple monitors, to represent exposure across the city for a given day.

### Study design

Our study utilized a time-stratified case-crossover design described by Schwartz [35]. This design was an adaptation of Maclure’s 1991 study [36]. This study design has been used to show a relationship between air pollution and myocardial infarction [37], ventricular arrhythmias [38], stroke [20], and asthma [39]. In a case-crossover study each patient serves as both the case and the control. Cases are identified by the date of hospital admission with UGIB secondary to PUD. Pollutant levels for each index date were matched and compared to a series of referent dates based on a time-stratified design. In this approach the referent dates selected were the same days of the week within the same month and year as the date of hospitalization. Bidirectional control sampling is valid because the effect of the event (i.e. UGIB secondary to PUD) does not influence air pollution levels [35]. Time stratified design adjusts for day of the week and seasonal effects. A detailed review of the performance of several different strategies to select control intervals within a case-crossover design showed in simulation models that the time-stratified approach was the best method to select control periods [40]. The measures of association generated from these comparisons rely on contrasts within-individuals and therefore, the design of the study is able to control for individual-level factors that are invariant (e.g. genetic factors, colonization with *Helicobacter pylori*) during the month where case and control intervals were identified.

### Data analysis

Descriptive statistics comparing air pollutant levels and patient characteristics between the two cities were generated using the χ^2^ or Mood’s median test. We examined the effect of each air pollutant (SO_2_, NO_2_, CO, O_3_, PM_10_ and PM_2.5_) individually. Conditional logistic regression was used to adjust for temperature and relative humidity when modeling the effects of air pollution. Odds ratios (OR) with 95 % confidence intervals (CI) were calculated to examine the incidence of UGIB secondary to PUD with each IQR increase in the pollutants. The IQRs were based on the daily levels for each pollutant throughout the entire study period and pooled between Calgary and Edmonton. Exposure to air pollution was measured using various time intervals: on the same day (0-day), previous day (1-day lag), and averages over 3, 5, and 7 days prior to event or referent days. In secondary *post hoc* analyses, we stratified all of our analyses by age (above and below 70 years, which was the median age of our population), sex (male versus female) and season (summer, fall, winter, and spring). We used an alpha value of 0.05 to define statistical significance. All statistical analyses were performed using SAS version 9.3.

The study was approved by the Conjoint Health Research Ethics Board at the University of Calgary. Informed consent was waived by the ethics board because patient data was anonymized.

## Results

We identified 1374 and 1159 incident cases of UGIB secondary to PUD in Calgary and Edmonton, respectively. Baseline characteristics stratified by city are described in Table 1. The proportion of perforated PUD was higher in Edmonton (3.4 %) as compared to Calgary (2.0 %) (*p <* 0.05). Also, Calgary residents suffering from UGIB secondary to PUD were less likely to have comorbidities (40.9 %) as compared to Edmonton (55.3 %) (*p <* 0.001). The median and interquartile range (IQR) for the air pollutants for Calgary, Edmonton, and pooled across cities are provided in Table 2.

**Table 1:** Characteristics of hospitalized patients with UGIB secondary to PUD from 2004 to 2010

Variable	Overall	Calgary	Edmonton	*P*-value
*Patients (%)*	2523	1374 (54.5)	1149 (45.5)	
*Median Age in Years (IQR)*	70.0 (54.0–80.0)	68.0 (53.0–80.0)	72.0 (56.0–80.0)	0.1
*Sex (%)*				0.9
Male	1561 (61.9)	851 (61.9)	710 (61.8)	
Female	962 (38.1)	523 (38.1)	439 (38.2)	
*Number of Comorbidities (%)*				<0.001
0	1326 (52.6)	812 (59.1)	514 (44.7)	
1	495 (19.6)	229 (16.7)	266 (23.2)	
2	289 (11.5)	138 (10.0)	151 (13.1)	
3	170 (6.7)	81 (5.9)	89 (7.8)	
4	101 (4.0)	48 (3.5)	53 (4.6)	
5	59 (2.3)	24 (1.8)	35 (3.1)	
≥6	83 (3.3)	42 (3.1)	41 (3.6)	
*Season (%)*				0.002
Autumn	635 (25.2)	314 (22.9)	321 (27.9)	
Spring	621 (24.6)	372 (27.0)	249 (21.7)	
Summer	659 (26.1)	369 (26.9)	290 (25.2)	
Winter	608 (24.1)	319 (23.2)	289 (25.2)	
*Perforated PUD (%)*				0.03
No	2456 (97.3)	1346 (98.0)	1110 (96.6)	
Yes	67 (2.7)	28 (2.0)	39 (3.4)

**Table 2:** Distribution of daily pollution concentrations from 2004 to 2010 and reported as median (25^th^–75^th^ percentiles)

Pollutant	Overall	Calgary	Edmonton	*P*-value
O_3_ (ppb)	21.7 (15.0–28.3)	19.3 (13.3–25.7)	23.7 (17.5–30.9)	0.2
SO_2_ (ppb)	1.0 (0.9–1.8)	1.0 (1.0–2.0)	1.1 (0.8–1.6)	<0.001
CO (ppm)	0.3 (0.2–0.4)	0.4 (0.3–0.4)	0.3 (0.2–0.4)	<0.001
NO_2_ (ppb)	12.7 (7.6–19.0)	17.3 (13.0–23.5)	7.9 (5.6–12.1)	<0.001
PM_2.5_ (μg/m^3^)	5.3 (3.5–7.7)	6.0 (4.3–8.7)	4.6 (3.1–6.6)	0.2
PM_10_ (μg/m^3^)	18.1 (13.0–27.0)	22.0 (15.0–32.0)	15.8 (12.0–21.8)	<0.001

*PPB* parts per billion, *PPM*, parts per million, *SO*_*2*_ sulfur dioxide, *NO*_*2*_ nitrogen dioxide, *CO* carbon monoxide, *O*_*3*_ ozone, *PM*_*10*_ particulate matter with aerodynamic diameter of ≤10 μm, *PM*_*2.5*_ particulate matters with aerodynamic diameter of ≤2.5 μm

### Discovery cohort (Calgary)

The results found using the discovery cohort in Calgary are reported in Table 3. Pollutant concentrations were not associated with developing UGIB secondary to PUD when the exposure period was on the same day or the day prior to being hospitalized. However, the incidence of UGIB secondary to PUD was inversely associated with NO_2_, PM_2.5_, and PM_10_ when accounting for average concentration over 3, 5, and/or 7 days.

**Table 3:** Association between exposure to air pollutants and hospitalization with UGIB secondary to PUD among patients residing in Calgary (*n =* 1374) and Edmonton (*n =* 1149)

Time interval	O_3_	SO_2_	NO_2_	CO	PM_2.5_	PM_10_
Discovery cohort (Calgary)						
0-day	1.14 (0.94–1.38)	0.92 (0.73–1.15)	0.94 (0.81–1.08)	0.95 (0.84–1.07)	0.92 (0.81–1.05)	1.01 (0.93–1.09)
1-day lag	0.85 (0.70–1.02)	1.02 (0.81–1.29)	1.01 (0.88–1.17)	1.05 (0.93–1.19)	0.93 (0.81–1.06)	0.95 (0.87–1.04)
3-day	0.95 (0.73–1.23)	0.88 (0.63–1.23)	0.92 (0.75–1.13)	0.96 (0.81–1.14)	0.85 (0.72–1.00)	0.94 (0.84–1.05)
5-day	1.07 (0.78–1.47)	0.87 (0.59–1.28)	0.68 (0.53–0.88)	0.82 (0.67–1.01)	0.75 (0.61–0.90)	0.87 (0.75–1.00)
7-day	1.06 (0.74–1.52)	0.90 (0.58–1.39)	0.67 (0.51–0.89)	0.85 (0.68–1.06)	0.78 (0.63–0.97)	0.88 (0.75–1.03)
Replication cohort (Edmonton)						
0-day	0.99 (0.79–1.24)	1.01 (0.85–1.20)	1.11 (0.90–1.36)	1.05 (0.90–1.22)	1.01 (0.90–1.14)	0.97 (0.84–1.13)
1-day lag	0.94 (0.75–1.17)	0.97 (0.80–1.17)	1.05 (0.86–1.28)	1.02 (0.88–1.19)	0.98 (0.86–1.11)	0.87 (0.74–1.01)
3-day	0.95 (0.72–1.27)	0.99 (0.76–1.29)	1.02 (0.79–1.33)	1.02 (0.83–1.24)	0.94 (0.80–1.10)	0.83 (0.68–1.00)
5-day	1.06 (0.77–1.46)	0.91 (0.66–1.26)	0.88 (0.65–1.18)	0.94 (0.74–1.18)	0.88 (0.73–1.06)	0.79 (0.64–0.99)
7-day	1.06 (0.75–1.50)	0.89 (0.61–1.29)	0.82 (0.58–1.15)	0.90 (0.69–1.17)	0.86 (0.70–1.06)	0.79 (0.61–1.01)

^*a*^*SO*_*2*_ sulfur dioxide, *NO*_*2*_ nitrogen dioxide, *CO* carbon monoxide, *O*_*3*_ ozone, *PM*_*10*_ particulate matter with aerodynamic diameter of ≤10 μm, *PM*_*2.5*_ particulate matters with aerodynamic diameter of ≤2.5 μm

### Replication cohort (Edmonton)

When the analysis was repeated using the replication cohort in Edmonton, none of the statistically significant associations observed in Calgary were replicated (Table 3). The only significant association between UGIB secondary to PUD and any of the pollutants for all exposure durations was an inverse association with 5-day average PM_10_.

### Secondary analyses

Stratifying the results by age (above versus below 70 years) (Additional file 1: Table S1), sex (Additional file 1: Table S2), and season (Additional file 1: Table S3) did not yield significant associations in Calgary that were replicated in Edmonton.

## Discussion

Based on emerging evidence that air pollution is associated with other gastrointestinal disorders [23–27], we hypothesized that acute exposure to air pollution triggered an UGIB secondary to PUD. Air pollutants have been shown to increase intestinal permeability, alter the gut microbiome, and promote inflammation, which may contribute towards gastric or duodenal bleeding [28–31]. However, our discovery and replication cohorts did not consistently demonstrate statistically significant associations between acute exposure to air pollutants and UGIB secondary to PUD. Furthermore, in Calgary we identified several statistically significant negative associations between air pollutants and UGIB secondary to PUD; however, these findings were not reproduced in our replication cohort in the city of Edmonton. Thus, acute exposure to air pollution does not appear to increase the risk of developing an UGIB secondary to PUD.

In the discovery cohort from Calgary, NO_2_, PM_2.5_, and PM_10_, over some lag periods were inversely associated with UGIB secondary to PUD. The inverse association may suggest that residual confounders may not have been addressed in our analysis. Additionally, the inverse association may be false positives due to multiple comparison error. Studies evaluating the effects of acute exposure to air pollution are subject to multiple comparisons bias because the associations are often assessed with multiple pollutants (e.g. SO_2_, NO_2_, CO, O_3_, PM_10_, and PM_2.5_) and different exposure periods (e.g. same day, 1-day lag, and cumulative day averages). Thus, the risk of observing a statistically significant association by chance is increased. This notion was supported by the observation that none of the associations observed in the discovery cohort were confirmed in the replication cohort.

The cities of Calgary and Edmonton are comparable because they share similarities in demographics, healthcare delivery, and the administrative data was collected by the same agency [41]. However, several differences were observed between the cities. For example, the median daily levels of SO_2_, CO, NO_2_ and PM_10_ were significantly higher in Calgary than in Edmonton. Differences in the siting characteristics of the monitor stations in the two cities (e.g. nearness to traffic) may also cause the two cities to experience different amounts exposure misclassification. Additionally, the patient characteristics of the UGIB secondary to PUD patients differed between cities. While patients were similar in age and sex distributions, patients in Edmonton had more comorbidities and were more likely to present with a perforation.

We carried out secondary analyses to evaluate whether the effect of air pollution on UGIB secondary to PUD was influenced by age, sex, and season. Overall, these stratified analyses were consistent with our primary analyses showing predominantly null associations that lacked replication. Because administrative databases lack many clinically relevant variables, we were not able to explore differences among other patient characteristics such as disease severity at presentation, *Helicobacter pylori* status, and use of NSAIDs. While these factors are not known to be influenced by air pollution exposure, we were not able to specifically evaluate PUD that was not caused by NSAIDs and *Helicobacter pylori*.

Our analysis relied on an administrative healthcare database to identify patients with UGIB secondary to PUD. We used a previously validated ICD-10 case-definition of UGIB secondary to PUD to identify cases [1]. The positive predictive value of our case definition was 85 %, which means that it is estimated that 15 % of our study population did not have an UGIB secondary to PUD [1]. Misclassification error associated with our case-definition may have influenced the effect on our risk estimates.

Several additional limitations of our study should be considered. We utilized a case-crossover study, which limited the observations to acute exposures in patients who have developed UGIB secondary to PUD. Furthermore, we assigned air pollution exposures from city-wide measurements from fixed monitoring stations that do not capture personal or household level exposure. However, several validation and simulation studies have supported this approach in studying the health effects of air pollution [35, 42]. The case-crossover study design controls for potential confounders that do not acutely vary across time (e.g. *Helicobacter pylori* status). However, time-dependent risk factors of PUD (e.g. NSAID use may vary daily) may have not been controlled. Unfortunately, our data sources do not track information regarding NSAID use or *Helicobacter pylori* status, making us unable to stratify by *Helicobacter pylori*, NSAID, or idiopathic PUD. Also, air pollution levels in Calgary and Edmonton are relatively low when compared to other regions in the world. Low levels of air pollution may reduce the variability over short periods of time that are necessary to detect health effects. However, similarly designed air pollution studies conducted in Calgary and Edmonton have consistently demonstrated significant associations for other health states [20, 25, 26, 39]. Finally, our study was limited to two cities in Alberta and thus, our findings may not necessarily be generalizable to other regions with different air pollution levels and patient profiles of UGIB secondary to PUD.

## Conclusions

Neither our discovery or replication cohort found an association between acute air pollution exposure and the development of UGIB secondary to PUD. Our discovery cohort produced inverse associations across several pollutants and different exposure periods. However, these findings were not observed in our replication cohort. Thus, the findings may be due to unmeasured confounders and/or spurious associations.

## Additional file

Additional file 1: Table S1. Association between exposure to air pollutants and hospitalization with UGIB secondary to PUD among patients residing in Calgary and Edmonton stratified by sex. (DOCX 31 kb)

## Abbreviations

UGIB: Upper gastrointestinal bleeding
PUD: Peptic ulcer disease
OR: Odds ratio
CI: Confidence interval
NSAIDs: Non-steroidal anti-inflammatory drugs
DAD: Discharge abstract databases
ICD-10: International Classifications of Diseases version 10
SO_2_: Sulfur dioxide
NO_2_: Nitrogen dioxide
CO: Carbon monoxide, O_3_, ozone
PM_10_: Particulate matter with aerodynamic diameter of ≤10 μm
PM_2.5_: Particulate matter with aerodynamic diameter of ≤2.5 μm, IQR, interquartile range
