# Supplementary material for: Upper gastrointestinal bleeding due to peptic ulcer disease is not associated with air pollution: a case-crossover study
Source: BMC Gastroenterol. 2015 Oct 14;15:131. doi: 10.1186/s12876-015-0363-6 (PMC4604641; doi:10.1186/s12876-015-0363-6)
Supplement: Additional file 1: Table S1. — Association between exposure to air pollutants and hospitalization with UGIB secondary to PUD among patients residing in Calgary and Edmonton stratified by sex. (DOCX 31 kb) [file 12876_2015_363_MOESM1_ESM.docx]

Table S1: Association between exposure to air pollutants and hospitalization with UGIB secondary to PUD among patients residing in Calgary and Edmonton stratified by sex.

| **Time Interval** | **O_3_** | **SO_2_** | **NO_2_** | **CO** | **PM_2.5_** | **PM_10_** |
| --- | --- | --- | --- | --- | --- | --- |
| **Females** | | | | | | |
| **Discovery Cohort (Calgary)** | | | | | | |
| 0-day | 1.17 (0.85-1.59) | 0.94 (0.65-1.36) | 0.81 (0.63-1.04) | 0.89 (0.72-1.10) | 0.85 (0.68-1.06) | 1.03 (0.90-1.18) |
| 1-day lag | 0.79 (0.58-1.09) | 0.92 (0.63-1.34) | 0.90 (0.70-1.15) | 0.95 (0.77-1.18) | 0.85 (0.68-1.07) | 0.93 (0.80-1.07) |
| 3-day | 0.98 (0.64-1.50) | 0.83 (0.49-1.43) | 0.70 (0.50-0.99) | 0.83 (0.62-1.11) | 0.79 (0.60-1.03) | 0.96 (0.80-1.16) |
| 5-day | 1.17 (0.70-1.97) | 1.02 (0.54-1.92) | 0.50 (0.33-0.77) | 0.71 (0.49-1.03) | 0.73 (0.53-1.00) | 0.94 (0.75-1.18) |
| 7-day | 1.28 (0.71-2.29) | 1.16 (0.57-2.36) | 0.55 (0.34-0.86) | 0.77 (0.52-1.13) | 0.84 (0.60-1.17) | 1.01 (0.78-1.31) |
| **Replication Cohort (Edmonton)** | | | | | | |
| 0-day | 1.29 (0.90-1.86) | 1.11 (0.87-1.40) | 1.05 (0.76-1.45) | 0.98 (0.77-1.25) | 1.11 (0.92-1.34) | 1.02 (0.80-1.29) |
| 1-day lag | 1.25 (0.87-1.80) | 1.19 (0.91-1.56) | 0.96 (0.69-1.34) | 0.90 (0.70-1.16) | 1.07 (0.89-1.30) | 0.86 (0.67-1.10) |
| 3-day | 1.31 (0.84-2.05) | 1.32 (0.91-1.92) | 0.94 (0.61-1.44) | 0.87 (0.63-1.22) | 1.07 (0.83-1.37) | 0.86 (0.63-1.17) |
| 5-day | 1.52 (0.91-2.53) | 1.18 (0.73-1.88) | 0.72 (0.43-1.20) | 0.77 (0.52-1.15) | 0.91 (0.68-1.22) | 0.76 (0.53-1.10) |
| 7-day | 1.45 (0.83-2.52) | 1.02 (0.57-1.79) | 0.66 (0.38-1.17) | 0.71 (0.45-1.11) | 0.86 (0.62-1.19) | 0.78 (0.52-1.18) |
| **Males** | | | | | | |
| **Discovery Cohort (Calgary)** | | | | | | |
| 0-day | 1.12 (0.88-1.43) | 0.90 (0.67-1.21) | 1.01 (0.84-1.22) | 0.98 (0.84-1.14) | 0.96 (0.82-1.13) | 0.99 (0.89-1.10) |
| 1-day lag | 0.88 (0.70-1.12) | 1.09 (0.81-1.47) | 1.08 (0.90-1.30) | 1.10 (0.95-1.28) | 0.98 (0.83-1.15) | 0.97 (0.86-1.08) |
| 3-day | 0.93 (0.67-1.29) | 0.91 (0.60-1.39) | 1.08 (0.84-1.40) | 1.04 (0.85-1.28) | 0.89 (0.73-1.09) | 0.93 (0.80-1.07) |
| 5-day | 1.02 (0.68-1.52) | 0.79 (0.48-1.29) | 0.80 (0.59-1.10) | 0.88 (0.68-1.13) | 0.76 (0.59-0.97) | 0.82 (0.69-0.99) |
| 7-day | 0.95 (0.61-1.50) | 0.77 (0.44-1.34) | 0.76 (0.53-1.07) | 0.89 (0.67-1.16) | 0.74 (0.56-0.98) | 0.80 (0.65-0.99) |
| **Replication Cohort (Edmonton)** | | | | | | |
| 0-day | 0.84 (0.63-1.12) | 0.92 (0.71-1.18) | 1.14 (0.88-1.48) | 1.09 (0.90-1.33) | 0.95 (0.81-1.11) | 0.94 (0.77-1.14) |
| 1-day lag | 0.77 (0.58-1.03) | 0.81 (0.62-1.06) | 1.09 (0.85-1.41) | 1.10 (0.91-1.33) | 0.92 (0.77-1.09) | 0.88 (0.72-1.07) |
| 3-day | 0.77 (0.53-1.12) | 0.75 (0.51-1.10) | 1.06 (0.75-1.47) | 1.10 (0.85-1.42) | 0.86 (0.70-1.06) | 0.81 (0.63-1.04) |
| 5-day | 0.86 (0.57-1.30) | 0.73 (0.47-1.15) | 0.95 (0.65-1.38) | 1.03 (0.76-1.38) | 0.85 (0.67-1.08) | 0.80 (0.61-1.06) |
| 7-day | 0.88 (0.56-1.39) | 0.80 (0.49-1.32) | 0.91 (0.59-1.40) | 1.02 (0.73-1.43) | 0.86 (0.66-1.12) | 0.78 (0.57-1.07) |

^a^SO_2_ - sulfur dioxide; NO_2_ - nitrogen dioxide; CO - carbon monoxide; O_3_ – ozone; PM_10_ - particulate matter with aerodynamic diameter of ≤10 µm; PM_2.5_ - particulate matters with aerodynamic diameter of ≤2.5 µm.

Table S2: Association between exposure to air pollutants and hospitalization with UGIB secondary to PUD among patients residing in Calgary and Edmonton stratified by age group.

| **Time Interval** | **O_3_** | **SO_2_** | **NO_2_** | **CO** | **PM_2.5_** | **PM_10_** |
| --- | --- | --- | --- | --- | --- | --- |
| **Age < 70 years** | | | | | | |
| **Discovery Cohort (Calgary)** | | | | | | |
| 0-day | 1.21 (0.93-1.56) | 0.81 (0.59-1.11) | 0.84 (0.69-1.03) | 0.92 (0.77-1.10) | 0.90 (0.74-1.08) | 0.97 (0.86-1.09) |
| 1-day lag | 0.86 (0.66-1.11) | 0.83 (0.61-1.15) | 0.92 (0.76-1.13) | 1.00 (0.85-1.18) | 0.85 (0.71-1.02) | 0.95 (0.84-1.07) |
| 3-day | 0.98 (0.68-1.40) | 0.70 (0.45-1.10) | 0.80 (0.60-1.06) | 0.92 (0.73-1.17) | 0.74 (0.59-0.94) | 0.92 (0.78-1.09) |
| 5-day | 1.04 (0.67-1.61) | 0.67 (0.40-1.14) | 0.59 (0.42-0.84) | 0.81 (0.61-1.08) | 0.61 (0.46-0.81) | 0.88 (0.72-1.07) |
| 7-day | 1.13 (0.69-1.85) | 0.69 (0.38-1.24) | 0.59 (0.40-0.87) | 0.87 (0.64-1.17) | 0.64 (0.46-0.88) | 0.87 (0.69-1.09) |
| **Replication Cohort (Edmonton)** | | | | | | |
| 0-day | 1.02 (0.72-1.43) | 0.91 (0.69-1.19) | 1.36 (1.01-1.84) | 1.26 (1.00-1.59) | 1.08 (0.90-1.30) | 1.02 (0.82-1.27) |
| 1-day lag | 0.99 (0.70-1.40) | 0.93 (0.71-1.24) | 1.15 (0.85-1.55) | 1.09 (0.88-1.37) | 1.06 (0.87-1.28) | 0.92 (0.72-1.16) |
| 3-day | 1.05 (0.68-1.63) | 0.93 (0.63-1.37) | 1.24 (0.84-1.83) | 1.15 (0.85-1.56) | 1.03 (0.81-1.31) | 0.91 (0.68-1.21) |
| 5-day | 1.26 (0.77-2.05) | 0.90 (0.56-1.43) | 0.99 (0.63-1.53) | 0.99 (0.70-1.40) | 0.89 (0.67-1.17) | 0.84 (0.61-1.16) |
| 7-day | 1.34 (0.78-2.28) | 0.88 (0.51-1.51) | 0.81 (0.49-1.33) | 0.91 (0.61-1.35) | 0.81 (0.59-1.11) | 0.79 (0.55-1.14) |
| **Age ≥ 70 years** | | | | | | |
| **Discovery Cohort (Calgary)** | | | | | | |
| 0-day | 1.07 (0.81-1.42) | 1.05 (0.75-1.48) | 1.05 (0.85-1.30) | 0.98 (0.82-1.16) | 0.95 (0.78-1.14) | 1.03 (0.92-1.15) |
| 1-day lag | 0.84 (0.63-1.11) | 1.31 (0.93-1.84) | 1.13 (0.91-1.40) | 1.11 (0.94-1.33) | 1.03 (0.85-1.25) | 0.95 (0.84-1.08) |
| 3-day | 0.91 (0.62-1.34) | 1.17 (0.71-1.91) | 1.08 (0.80-1.46) | 1.00 (0.78-1.29) | 0.96 (0.77-1.21) | 0.95 (0.81-1.12) |
| 5-day | 1.09 (0.68-1.73) | 1.21 (0.68-2.15) | 0.81 (0.56-1.17) | 0.84 (0.61-1.14) | 0.89 (0.68-1.15) | 0.85 (0.70-1.04) |
| 7-day | 0.98 (0.59-1.65) | 1.23 (0.65-2.35) | 0.78 (0.52-1.17) | 0.82 (0.59-1.16) | 0.92 (0.69-1.24) | 0.88 (0.70-1.10) |
| **Replication Cohort (Edmonton)** | | | | | | |
| 0-day | 0.96 (0.71-1.30) | 1.10 (0.88-1.38) | 0.94 (0.71-1.24) | 0.93 (0.76-1.14) | 0.96 (0.82-1.13) | 0.94 (0.77-1.14) |
| 1-day lag | 0.88 (0.65-1.19) | 0.99 (0.77-1.29) | 0.99 (0.75-1.30) | 0.98 (0.80-1.20) | 0.92 (0.77-1.10) | 0.83 (0.68-1.02) |
| 3-day | 0.86 (0.59-1.26) | 1.06 (0.73-1.53) | 0.89 (0.62-1.27) | 0.93 (0.71-1.23) | 0.87 (0.70-1.08) | 0.77 (0.59-1.00) |
| 5-day | 0.90 (0.58-1.38) | 0.92 (0.59-1.44) | 0.82 (0.54-1.24) | 0.92 (0.67-1.27) | 0.89 (0.69-1.14) | 0.76 (0.56-1.03) |
| 7-day | 0.86 (0.54-1.37) | 0.87 (0.52-1.47) | 0.84 (0.52-1.35) | 0.91 (0.63-1.30) | 0.92 (0.69-1.21) | 0.79 (0.56-1.11) |

^a^SO_2_ - sulfur dioxide; NO_2_ - nitrogen dioxide; CO - carbon monoxide; O_3_ – ozone; PM_10_ - particulate matter with aerodynamic diameter of ≤10 µm; PM_2.5_ - particulate matters with aerodynamic diameter of ≤2.5 µm.

Table S3: Association between exposure to air pollutants and hospitalization with UGIB secondary to PUD among patients residing in Calgary and Edmonton stratified by season.

| **Time Interval** | **O_3_** | **SO_2_** | **NO_2_** | **CO** | **PM_2.5_** | **PM_10_** |
| --- | --- | --- | --- | --- | --- | --- |
| **Autumn** | | | | | | |
| **Discovery Cohort (Calgary)** | | | | | | |
| 0-day | 0.83 (0.54-1.28) | 0.59 (0.35-1.01) | 1.02 (0.72-1.44) | 1.02 (0.77-1.34) | 0.76 (0.56-1.04) | 0.85 (0.70-1.04) |
| 1-day lag | 0.72 (0.45-1.13) | 0.82 (0.48-1.39) | 0.93 (0.64-1.33) | 1.11 (0.82-1.49) | 0.87 (0.63-1.20) | 0.84 (0.68-1.03) |
| 3-day | 0.57 (0.30-1.06) | 0.66 (0.30-1.45) | 1.07 (0.65-1.75) | 1.27 (0.83-1.94) | 0.79 (0.54-1.16) | 0.85 (0.66-1.11) |
| 5-day | 0.49 (0.23-1.05) | 0.62 (0.24-1.64) | 0.82 (0.45-1.48) | 1.10 (0.66-1.81) | 0.67 (0.43-1.04) | 0.81 (0.60-1.11) |
| 7-day | 0.32 (0.13-0.79) | 0.91 (0.30-2.73) | 0.91 (0.46-1.82) | 1.28 (0.72-2.26) | 0.66 (0.39-1.10) | 0.89 (0.63-1.27) |
| **Replication Cohort (Edmonton)** | | | | | | |
| 0-day | 0.99 (0.79-1.24) | 1.01 (0.85-1.20) | 1.11 (0.90-1.36) | 1.05 (0.90-1.22) | 1.01 (0.90-1.14) | 0.97 (0.84-1.13) |
| 1-day lag | 0.94 (0.75-1.17) | 0.97 (0.80-1.17) | 1.05 (0.86-1.28) | 1.02 (0.88-1.19) | 0.98 (0.86-1.11) | 0.87 (0.74-1.01) |
| 3-day | 0.95 (0.72-1.27) | 0.99 (0.76-1.29) | 1.02 (0.79-1.33) | 1.02 (0.83-1.24) | 0.94 (0.80-1.10) | 0.83 (0.68-1.00) |
| 5-day | 1.06 (0.77-1.46) | 0.91 (0.66-1.26) | 0.88 (0.65-1.18) | 0.94 (0.74-1.18) | 0.88 (0.73-1.06) | 0.79 (0.64-0.99) |
| 7-day | 1.06 (0.75-1.50) | 0.89 (0.61-1.29) | 0.82 (0.58-1.15) | 0.90 (0.69-1.17) | 0.86 (0.70-1.06) | 0.79 (0.61-1.01) |
| **Spring** | | | | | | |
| **Discovery Cohort (Calgary)** | | | | | | |
| 0-day | 1.23 (0.87-1.74) | 0.96 (0.61-1.50) | 1.02 (0.78-1.34) | 1.06 (0.78-1.45) | 1.01 (0.77-1.34) | 1.12 (0.94-1.32) |
| 1-day lag | 1.08 (0.78-1.48) | 1.35 (0.88-2.08) | 0.97 (0.75-1.26) | 1.02 (0.75-1.37) | 1.10 (0.84-1.43) | 1.05 (0.89-1.25) |
| 3-day | 1.31 (0.84-2.04) | 1.22 (0.65-2.27) | 0.99 (0.70-1.42) | 0.92 (0.60-1.41) | 1.01 (0.71-1.42) | 1.09 (0.88-1.35) |
| 5-day | 1.64 (0.96-2.80) | 1.22 (0.58-2.58) | 0.81 (0.52-1.26) | 0.64 (0.36-1.13) | 0.87 (0.57-1.33) | 0.96 (0.73-1.27) |
| 7-day | 1.67 (0.93-3.03) | 1.29 (0.57-2.90) | 0.81 (0.50-1.31) | 0.62 (0.33-1.16) | 1.07 (0.68-1.69) | 0.97 (0.70-1.36) |
| **Replication Cohort (Edmonton)** | | | | | | |
| 0-day | 0.85 (0.55-1.32) | 1.14 (0.87-1.51) | 0.88 (0.59-1.33) | 0.96 (0.72-1.27) | 0.87 (0.67-1.12) | 0.86 (0.68-1.09) |
| 1-day lag | 1.19 (0.76-1.86) | 1.08 (0.79-1.50) | 0.69 (0.44-1.09) | 0.75 (0.55-1.03) | 0.72 (0.54-0.96) | 0.75 (0.58-0.97) |
| 3-day | 0.92 (0.52-1.63) | 1.30 (0.84-2.01) | 0.68 (0.38-1.21) | 0.75 (0.50-1.13) | 0.67 (0.48-0.94) | 0.66 (0.48-0.90) |
| 5-day | 1.07 (0.55-2.07) | 1.04 (0.58-1.85) | 0.54 (0.27-1.09) | 0.64 (0.38-1.06) | 0.64 (0.44-0.94) | 0.58 (0.41-0.84) |
| 7-day | 1.00 (0.48-2.08) | 0.99 (0.50-1.97) | 0.63 (0.28-1.42) | 0.67 (0.37-1.21) | 0.70 (0.46-1.07) | 0.57 (0.37-0.86) |
| **Summer** | | | | | | |
| **Discovery Cohort (Calgary)** | | | | | | |
| 0-day | 1.04 (0.71-1.53) | 1.21 (0.71-2.07) | 0.87 (0.52-1.44) | 0.75 (0.42-1.34) | 1.04 (0.79-1.37) | 0.96 (0.77-1.20) |
| 1-day lag | 0.70 (0.47-1.03) | 0.93 (0.54-1.59) | 0.92 (0.58-1.48) | 1.10 (0.64-1.88) | 0.93 (0.71-1.21) | 0.94 (0.76-1.17) |
| 3-day | 0.65 (0.38-1.12) | 0.82 (0.35-1.94) | 0.81 (0.42-1.57) | 0.82 (0.38-1.76) | 0.89 (0.63-1.24) | 0.87 (0.64-1.18) |
| 5-day | 0.78 (0.41-1.50) | 1.07 (0.38-3.06) | 0.49 (0.22-1.08) | 0.60 (0.24-1.47) | 0.87 (0.59-1.28) | 0.92 (0.66-1.30) |
| 7-day | 0.79 (0.37-1.66) | 0.79 (0.23-2.75) | 0.52 (0.21-1.27) | 0.65 (0.23-1.85) | 0.89 (0.58-1.36) | 0.96 (0.66-1.40) |
| **Replication Cohort (Edmonton)** | | | | | | |
| 0-day | 1.33 (0.84-2.11) | 1.29 (0.84-1.98) | 1.20 (0.77-1.87) | 0.96 (0.63-1.47) | 1.20 (0.85-1.68) | 1.21 (0.85-1.72) |
| 1-day lag | 1.13 (0.70-1.84) | 1.03 (0.69-1.54) | 1.06 (0.68-1.65) | 1.07 (0.72-1.60) | 1.10 (0.79-1.53) | 0.94 (0.66-1.33) |
| 3-day | 1.30 (0.72-2.33) | 1.09 (0.59-2.00) | 1.05 (0.61-1.82) | 0.96 (0.57-1.62) | 1.09 (0.69-1.70) | 0.97 (0.62-1.53) |
| 5-day | 1.31 (0.67-2.54) | 0.94 (0.45-1.97) | 0.97 (0.54-1.76) | 0.91 (0.52-1.59) | 0.97 (0.58-1.63) | 0.96 (0.56-1.62) |
| 7-day | 1.31 (0.65-2.61) | 1.04 (0.44-2.48) | 0.77 (0.41-1.44) | 0.76 (0.42-1.37) | 0.79 (0.45-1.39) | 0.84 (0.47-1.52) |
| **Winter** | | | | | | |
| **Discovery Cohort (Calgary)** | | | | | | |
| 0-day | 1.51 (1.03-2.22) | 1.02 (0.67-1.54) | 0.86 (0.69-1.08) | 0.92 (0.78-1.08) | 0.89 (0.63-1.24) | 1.02 (0.90-1.16) |
| 1-day lag | 0.87 (0.58-1.29) | 0.98 (0.64-1.50) | 1.15 (0.91-1.46) | 1.07 (0.91-1.25) | 0.87 (0.60-1.27) | 0.93 (0.80-1.08) |
| 3-day | 1.36 (0.78-2.40) | 0.85 (0.47-1.52) | 0.90 (0.65-1.27) | 0.95 (0.76-1.18) | 0.72 (0.45-1.14) | 0.89 (0.72-1.08) |
| 5-day | 1.64 (0.80-3.38) | 0.81 (0.43-1.53) | 0.69 (0.45-1.05) | 0.87 (0.67-1.13) | 0.46 (0.25-0.87) | 0.80 (0.63-1.02) |
| 7-day | 1.96 (0.85-4.54) | 0.84 (0.41-1.73) | 0.64 (0.40-1.02) | 0.88 (0.66-1.16) | 0.41 (0.19-0.86) | 0.77 (0.58-1.02) |
| **Replication Cohort (Edmonton)** | | | | | | |
| 0-day | 1.04 (0.59-1.81) | 0.99 (0.70-1.38) | 1.00 (0.43-2.33) | 1.26 (0.72-2.20) | 1.04 (0.85-1.28) | 0.94 (0.66-1.32) |
| 1-day lag | 0.65 (0.38-1.10) | 0.95 (0.65-1.39) | 0.82 (0.33-2.04) | 0.92 (0.49-1.70) | 1.03 (0.81-1.30) | 0.82 (0.57-1.18) |
| 3-day | 0.87 (0.44-1.72) | 0.96 (0.56-1.64) | 0.52 (0.14-1.89) | 0.88 (0.41-1.92) | 0.99 (0.73-1.35) | 0.75 (0.47-1.20) |
| 5-day | 1.04 (0.48-2.25) | 0.98 (0.52-1.85) | 0.21 (0.04-1.04) | 0.62 (0.25-1.55) | 0.87 (0.60-1.25) | 0.64 (0.37-1.09) |
| 7-day | 1.06 (0.44-2.53) | 0.88 (0.43-1.80) | 0.14 (0.02-0.86) | 0.59 (0.21-1.61) | 0.79 (0.52-1.19) | 0.61 (0.34-1.10) |

^a^SO_2_ - sulfur dioxide; NO_2_ - nitrogen dioxide; CO - carbon monoxide; O_3_ – ozone; PM_10_ - particulate matter with aerodynamic diameter of ≤10 µm; PM_2.5_ - particulate matters with aerodynamic diameter of ≤2.5 µm.
